# Results of Second-Look Laparotomy in Advanced Ovarian Cancer: One Single Center Experience

**DOI:** 10.5402/2012/849518

**Published:** 2012-10-16

**Authors:** Tarak Damak, Riadh Chargui, Jamel Ben Hassouna, Monia Hechiche, Khaled Rahal

**Affiliations:** Department of Surgical Oncology, Salah Azaiz Institute, 1029 Tunis, Tunisia

## Abstract

*Objective*. The goal of the study was to analyse the results of 85 cases of second-look laparotomy (SLL) and explore the influence of this procedure on survival. *Patients and Methods*. We reviewed retrospectively 85 cases of SLL collected and treated in our institute between 1994 and 2003. *Results*. Complete pathologic response (CPR) was 25.8%, microscopic disease (Rmicro) was 38.8%, and macroscopic disease (Rmacro) was 35.4%. In patients with negative SLL results, disease recurrence was diagnosed in 41%. The 3- and 5-year overall survival rates for the entire population were 91% and 87%, respectively. The 3- and 5-year disease-free survivals were, respectively, 76.3% and 58.5% in negative SLL versus 55.7% and 16% in positive SLL. The difference between the group of patients with complete response (76%) and the patients with residual microscopic disease (72%) was not significant. The tumoral residuum after initial surgery was the only prognostic factor influencing significantly the disease-free survival. On Cox regression model analysis, only initial tumoral residuum (*P* = 0.04) and tumoral residuum after SLL (*P* = 0.02) were independent prognostic factors for survival. *Conclusions*. The most important advantage of SLL is the early detection of recurrence and thus the early administration of consolidation treatment resulting in a better prognosis.

## 1. Introduction

Although second-look laparotomy (SSL) has been used in the management of ovarian cancer for over four decades, its current clinical use is limited. Over 50% to 70% of patients with a clinical complete response are noted to have disease at the time of SLL [1, 2]. The lack of accuracy of clinical examination, CA125 serum level, and noninvasive imaging methods for detecting residual disease explains the previous results [3, 4]. 

Although findings at SLL have some prognostic significance, there is no definitive evidence that the patients undergoing SLL have improved survival [5]. 

Although SLL permits to detect residual disease as early as possible, there is no improved survival finally approved due to the lack of the efficacy of salvage and consolidation regimens. 

We try to clarify in this present series the role of SLL in the management of advanced epithelial ovarian cancer in our institute.

## 2. Patients and Methods

This is a retrospective study about 85 cases of malignant ovarian tumors having an SLL and collected in the Department of Surgical Oncology at Salah Azaiz Institute of Tunis From January 1994 to December 2003. The inclusion criteria were a complete clinical remission after accomplishing the first-line chemotherapy. The clinical remission was assessed using the following criteria: no abnormalities in the physical and gynecologic examination, CA 125 serum concentration up to 351 U/mL, and no changes in available imaging procedures. Patients with other malignancies were excluded. 

The aim of SLL was either to confirm a clinical diagnosis of complete or partial response or to perform further tumor debulking in responders. SLL consisted of inspection and multiple biopsies of suspected lymph nodes, peritoneal surfaces, liver abnormalities, diaphragm scrapings, and cytological evaluation of ascitic fluid if present, otherwise peritoneal washings. A pathological complete response (PCR) was defined at SLL as no persistent macroscopic or microscopic disease. A microscopic partial response was defined as no visible tumor but positive histology and/or cytology in patients with macroscopic tumor after primary laparotomy.

Complete data regarding survival was obtained in all patients and analysed using SPSS 19 software for windows. Survival times were calculated from the time of diagnosis until the date of death or last contact. Actuarial survival curves were obtained using the Kaplan-Meier method and comparison of survival was performed with the log-rank and chi-squared tests. In this study, a *P* value of 0.05 or less was considered significant.

## 3. Results

Data regarding the analysed population are summarized in Table 1. 

The mean delay between the end of chemotherapy and the SLL was 10.6 weeks (3–47). Overall perioperative morbidity was 12.3%, intraoperative complication rate was 2.5%, and postoperative complication was 9%. There was no death due to perioperative complications in the analyzed population.

In the group of the 85 patients undergoing SLL we obtained the following results (Table 2).

The lymph nodes dissection was carried out in 30 patients (35.2%) during SLL procedure. In 18 cases (21.1%) with negative SLL and in 12 cases during positive SLL findings (14.1%). The mean number of removed lymph nodes was 20 (1–49). The mean number of involved lymph nodes was 3.4 (1–8).

**Figure 1:**
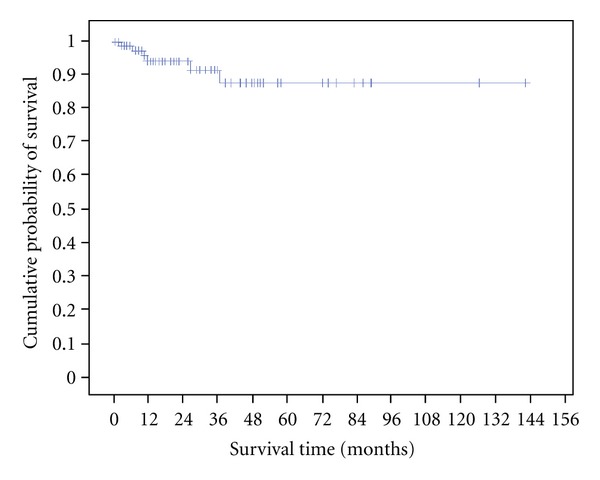
Overall survival.

The 3- and 5-year overall survival rates for the entire population were 91% and 87%, respectively. The mean survival time was 126 months (Figure 1). The high rate of survival is probably explained by the number of patients lost in contact. 

**Figure 2:**
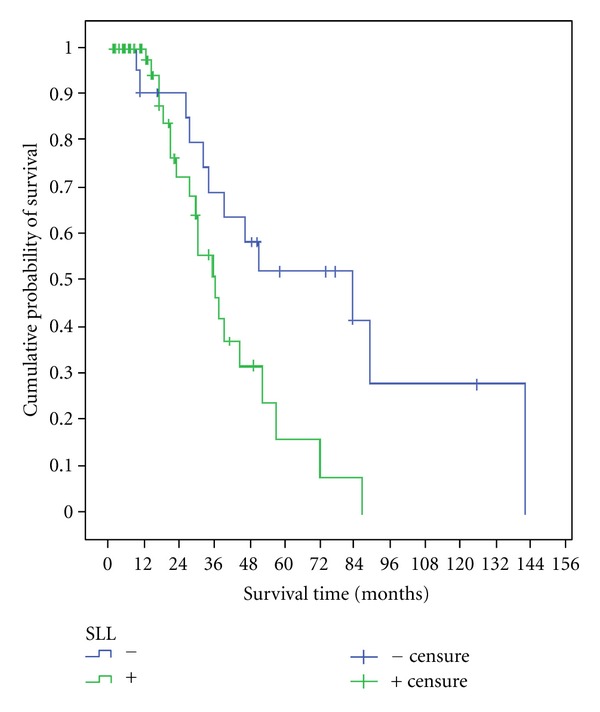
Disease-free survival.

The 3- and 5-year disease-free survival were, respectively, 76.3% and 58.5% in negative SLL versus 55.7% and 16% in positive SLL. The mean disease-free survival times were 83 and 36 months in the two groups, respectively (Figure 2).

Among the 22 patients with negative findings on SLL, nine (41%) patients recurred with a mean delay of 156 months. 

We compared survival curves in separate groups of patients depending on the following prognostic factors (see Table 3).

The difference between the group of patient with complete response (76%) and the patient with residual microscopic disease (72%) was not significant. 

The tumoral residuum after initial surgery was the only prognostic factor influencing significantly the disease-free survival. 

Multivariate analysis included the statistically significant prognostic factors for overall survival on univariate analysis. Only initial tumoral residuum (*P* = 0.04) and tumoral residuum after SLL (*P* = 0.02) were independent prognostic factors for survival.

## 4. Discussion

There is a common and serious misunderstanding about the purpose of the second-look surgery. Most authors discuss and analyse as if its primary goal is therapeutic, while in the majority of cases the primary function of second-look surgery is that of a diagnostic test [6]. Such exploratory surgery, whether by the open or laparoscopic approach, has been and remains the single most specific and sensitive means available for determining the status of the cancer before, during, or after chemotherapy [6–8].

There is not a shadow of doubt that second-look surgery is the most reliable “test” for the status of ovarian cancer. It is not perfect, but it is substantially superior to CT scan, MRI, physical examination, serum CA 125, and even to FDG-PET/CT [9, 10]. 

If the therapeutic value of SLL is not really proven, this is because of the lack of efficacious consolidation therapy [6]. 

In our country, we have not yet the FDG-PET and we think that SLL has yet a role to play in the management of ovarian cancer regarding not only its diagnostic value but also its therapeutic value. 

A comparison between the published series of SLL procedures is difficult because of the different distribution of prognostic factors. Usually during SLL persistent disease is found in 23–70% of cases [11, 12], in our series this rate was 74.2%. These results show that SLL is so far the most accurate method of evaluating response for first-line therapy in the group of patients with complete clinical remission. In our study, the outcome in separate groups of patients after SLL could indirectly confirm the potential benefits of early treatment of patients with persistent disease. The relationship between survival rates in patients depending on SLL results observed in our study is comparable to data in the literature [13–15]. 

There is no difference in survival between the group of patient with negative SLL findings compared with that with microscopic disease; this is probably explained by the early introduction of second-line or consolidation chemotherapy as it was found by Sawicki et al. [13].

These data suggest as for Sawicki et al. that early administration of therapy, based on the results of SLL, can result in improved outcome [13]. Further randomized studies are required to approve or not these findings. 

Furthermore, apart from the diagnostic value of SLL there is a therapeutic value especially for the patients who did not have lymph node dissection or incomplete cytoreductive surgery during the first laparotomy.In our study, we found a mean number of involved lymph node of 3.4 on SLL.

Another limitation of the SLL is the fact that a large number of patients will develop recurrence after a negative result of SLL. In our study, recurrence rate was 41%. In other studies analyzing second-look procedures, the recurrence rate ranged from 19.5 to 56.8% [8, 13, 16], such variance is due to the different distribution of prognostic factors. 

Despite the complete pathologic response on SLL, the recurrence rate is relatively high; this is may be explained by the aggressive biology of the tumor that is associated with a poor prognosis. Further researches are warranted to find the best consolidation treatment for this group of patients, and the SLL in this case will take a further place because it remains the best exploratory procedure for the evaluation of the response to the treatment. 

## 5. Conclusion

The most important advantage of SLL is the early detection of recurrence and thus the early administration of consolidation treatment. The SLL occupies an important role in the evaluation of new therapeutic agents because it remains the most accurate exam for the exploration of residual disease. Further prospective, randomized, and controlled trials are needed to evaluate the various therapies available. Lack of the confirmed efficacy of SLL is caused by ineffective second-line treatment. It is probable that if new drugs are developed in the future, the use of SLL will be discussed once again.

## Figures and Tables

**Table 1:** Characteristics of patients (*n* = 85).

Mean age	50 years	21–70
Mean CA 125 before SLL	1879 UI/mL	21–31599
FIGO stage	II	13 (15%)
	III	63 (74%)
	IV	9 (11%)
Histological types	Serous	57 (67.1%)
	Endometrial	11 (12.9%)
	Mucinous	4 (4.7%)
	Mixed	4 (4.7%)
	Unclassified types	9 (10.6%)
Grade	I	3 (3.5%)
	II	12 (14.1%)
	III	14 (16.5%)
Optimal cytoreduction after primary surgery (R < 1 cm)	FIGO II–IV	55 (64.7%)
Type of primary chemotherapy	Cisplatin-cyclophosphamide	68 (70%)
	Carboplatin-paclitaxel	17 (30%)
SLL duration	120.1 min	45–240 min
Hospital stay after SLL	8.5 days	5–26

**Table 2:** Second-look laparotomy results.

Residuum at SLL	Total	Percentage (%)
Null	22	25.8
Microscopic	33	38.8
Macroscopic	30	35.4

Total	85	100

**Table 3:** Prognostic factors for overall survival.

Prognostic factors	5-year overall survival (%)	*P* value
Age		0.17
<50 years	93	
≥50 years	78.6	
Stage		0.4
II	77	
III–IV	53.3	
Histological type		0.6
Serous	68.7	
Mucinous	75	
Other	92	
Histological grade		**0.002 **
Grade I	85	
Grades II-III	33.2	
Initial tumoral residuum		**0.017**
Null	64.4	
<1 cm	0	
>1 cm	61	
Second look		**0.031**
Negative	90	
Positive	77.6	
Lymphadenectomy at SLL		0.067
yes	84.1	
No	76.9	
Residuum at SLL		**0.003**
Null	76	
Microscopic	72	
Macroscopic	30.7	

SLL: second-look laparotomy.
